# Peripheral Mitochondrial DNA Copy Number is Increased in Korean Attention-Deficit Hyperactivity Disorder Patients

**DOI:** 10.3389/fpsyt.2019.00506

**Published:** 2019-07-18

**Authors:** Johanna Inhyang Kim, Soo-Young Lee, Mira Park, Si Yeon Kim, Jae-Won Kim, Soon Ae Kim, Bung-Nyun Kim

**Affiliations:** ^1^Department of Psychiatry, Hanyang University Medical Center, Seoul, South Korea; ^2^Department of Pharmacology, School of Medicine, Eulji University, Daejeon, South Korea; ^3^Department of Preventive Medicine, School of Medicine, Eulji University, Daejeon, South Korea; ^4^Division of Child and Adolescent Psychiatry, Department of Psychiatry, Seoul National University College of Medicine, Seoul, South Korea

**Keywords:** attention-deficit hyperactivity disorder (ADHD), mitochondrial dysfunction, mitochondrial DNA (mtDNA), peroxisome-proliferator-activated receptor γ co-activator-1α,DNA methylation

## Abstract

The involvement of mitochondrial dysfunction in the pathophysiology of attention-deficit hyperactivity disorder (ADHD) has been suggested in several reports. Mitochondrial DNA (mtDNA) copy number as well as methylation of the D-loop region and peroxisome-proliferator-activated receptor γ co-activator-1α (*PPARGC1A*) are considered biomarkers for mitochondrial dysfunction. We compared the mtDNA copy number and methylation ratio of the D-loop region and *PPARGC1A* between ADHD patients and controls and also among ADHD subtypes. The present study included 70 subjects with ADHD and 70 age- and gender-matched healthy controls (HCs). We measured the relative mtDNA copy number in peripheral blood cells using quantitative polymerase chain reaction (qPCR), and the methylation ratio was measured using methylation-specific PCR (MSP) after bisulfite conversion. The relative mtDNA copy number was significantly higher in ADHD patients than in HCs (*p* = 0.028). The mtDNA methylation ratio of *PPARGC1A* was decreased in ADHD patients compared with HCs (*p* = 0.008). After adjusting for IQ level, only the mtDNA copy number differed between the ADHD and HCs (*p* = 0.01). There was a significant difference in the methylation ratio of *PPARGC1A* among ADHD subtypes. These results suggest the possible involvement of mitochondrial dysfunction in the pathophysiology of ADHD. Further large cohort studies investigating the correlation between clinical markers and biomarkers of mitochondrial dysfunction are warranted.

## Introduction

Attention-deficit hyperactivity disorder (ADHD) is a highly prevalent and persistent neurodevelopmental disorder characterized by developmentally inappropriate symptoms of inattention, hyperactivity, and/or impulsivity (1). ADHD has an estimated heritability of 76% (2) and appears to be a complex polygenic disorder influenced by various genetic and environmental factors. Most of the genetic studies on ADHD have focused on dopaminergic and noradrenergic genes (3); however, the findings of these studies are inconsistent and explain only a small proportion of the genetic factors in ADHD (3). Currently, the biological mechanism underlying ADHD pathophysiology is largely unknown (4).

Reportedly, mitochondrial dysfunction is involved in the pathogenesis of various psychiatric disorders (5–7). Mitochondria are specialized subcellular organelles that contribute to aerobic ATP generation through oxidative phosphorylation for energy metabolism (8). It also plays crucial roles in calcium signaling, which is involved in exocytosis, synaptic plasticity, and the generation of reactive oxygen species (ROS) in the brain (9, 10). Mitochondria have been implicated in multiple neurodevelopmental processes central to synaptopathies, including neuronal differentiation (11), process outgrowth (12), cortical migration (13), and synaptogenesis (14).

Several recent studies showed that mitochondrial dysfunction underlies ADHD pathogenesis. In one previous study, lower mitochondrial respiration, lower ATPase 6/8 transcripts levels, reduced mitochondrial complex V activity, loss of mitochondrial membrane potential, and elevated oxidative stress in ADHD cybrids were reported (15). Another study showed a significant association of mtDNA 10398 A/G polymorphism with ADHD in Korean children (10). In animal studies, inhibition of the mitochondrial respiratory chain in the brain was observed following administration of methylphenidate (16). However, to date, research regarding the role of mitochondria in the development of ADHD is very limited.

Mitochondria contain their own genome, i.e., the mitochondrial DNA (mtDNA), which encodes essential subunits of the respiratory chain wherein electrons are combined with oxygen to allow the flow of energy through mitochondria (17). MtDNA is highly prone to mutations caused by low-fidelity DNA polymerase activity (18), lack of protective histones, and continuous exposure to the mutagenic effects of oxygen radicals (19). MtDNA copy number is a strong biomarker for mitochondrial dysfunction, since it may be increased with mtDNA damage or mitochondrial dysfunction to compensate for mitochondrial energy metabolism (20). However, to date, changes in mtDNA copy number in ADHD patients have not been investigated.

The D-loop, 1,124 bp in size (positions 16024-576) is a non-coding region that acts as a promoter of both the heavy and light strands of mtDNA and contains essential transcription and replication elements (21). The D-loop region is also a hotspot for mtDNA alterations (21). The peroxisome-proliferator-activated receptor γ co-activator-1α (*PPARGC1A*) is a co-transcriptional regulation factor involved in the induction of mitochondrial biogenesis. It activates several transcription factors like the nuclear respiratory factor 1 and nuclear respiratory factor 2, which in turn activate mitochondrial transcription factor A (22). The latter drives the transcription and replication of mtDNA (23). The methylation ratio of these two regions could also be considered a biomarker of mitochondrial dysfunction.

In this study, the mtDNA copy number and methylation ratios of the D-loop region and *PPARGC1A* were compared between ADHD subjects and healthy controls (HCs). We used an age- and gender-matched sample to control for biases caused by age and gender. We hypothesized that 1) the mtDNA copy number would be increased and 2) the methylation ratios of the D-loop region and *PPARGC1A* would be decreased in the ADHD group, leading to increased gene expression to compensate for mitrochondrial dysfunction.

## Methods

### Participants

Kim et al. (24) provides a description of the detailed recruitment protocol of this study. The recruitment period of 6–17-year-old ADHD and HCs was August 2010 to February 2015. The participants from two studies with the same study protocol were combined in the final analysis. In the first study, 90 ADHD patients and 33 HCs were initially recruited, and 1 HC subject was excluded for reason of missing genetic data (25). Among the 191 ADHD patients and 78 HCs recruited in the second study, 18 ADHD and 11 HCs were excluded due to missing genetic data (26). Consequently, the final number of total subjects included was 263 ADHD patients and 99 HCs. As the ADHD and HC groups differed in age and gender distribution, we further matched the ADHD group to the HC group based on age and gender. This resulted in 70 participants each in the ADHD and HC groups.

ADHD patients were recruited from the outpatient clinic of Seoul National University Hospital. Board-certified child and adolescent psychiatrists confirmed psychiatric diagnoses according to the *Diagnostic and Statistical Manual of Mental Disorders*, *Fourth Edition* (DSM-IV) criteria using the Korean Kiddie Schedule for Affective Disorders and Schizophrenia, Present and Lifetime version (K-SADS-PL) (27, 28). Exclusion criteria for ADHD patients were as follows: intelligence quotient (IQ) below 70; a hereditary genetic disorder; current or past history of brain trauma, organic brain disorder, seizure, or any neurological disorder; autism spectrum disorder, communication disorder or learning disorder; schizophrenia or any other childhood-onset psychotic disorder; major depressive disorder or bipolar disorder; Tourette’s syndrome or a chronic motor/vocal tic disorder; and obsessive compulsive disorder or a history of methylphenidate treatment lasting over 1 year or having taken the drug within the past 4 weeks. The HC group consisted of youth free of any psychiatric diagnoses based on the K-SADS-PL interview. The exclusion criteria for the HC group was the same as that for the ADHD group, with an additional criterion of a diagnosis of ADHD. IQ was measured using the Korean Educational Developmental Institute’s Wechsler Intelligence Scale for Children (29).

We used the parent-report Korean version of the ADHD Rating Scale-IV (ADHD-RS) to assess the severity of ADHD symptoms (30). The ADHD-RS includes 18 items, each item rated on a scale from 0 to 3. Total scores range from 0 to 54, higher scores implicating a greater severity. Nine items correspond to inattention symptoms, and nine correspond to hyperactivity–impulsivity symptoms (31).

Written informed consent was obtained from legal guardians and adolescent participants; child participants provided verbal consent after thorough explanation of the study. All study protocols were reviewed by the Institutional Review Board of Seoul National University Hospital, Seoul, Korea.

### Measurement of mtDNA Copy Number

The mtDNA copy number was evaluated based on the ratio of mtDNA to nuclear DNA. The mtDNA and nuclear DNA were quantified based on the mitochondrial gene, cytochrome b (CYTB), and the single-copy nuclear pyruvate kinase (PK) gene, respectively. The relative amounts of mtDNA and nuclear DNA were measured using quantitative polymerase chain reaction (qPCR) with the primers reported by Yoo et al. (32). The primers for the CYTB gene were forward 5’-CACGATTCTTTACCTTTCACTTCATC-3’ and reverse 5’-TGATCCCGTTTCGTGCAAG-3’. The primers for the PK gene were forward 5’-AGCCCAAATGGCCTTGAAG-3’ and reverse 5’-AGAGACAGAATGCCAGTGAGCTT-3’. Genomic DNA (20 ng) was used as template per 10 µl reaction with IQ SYBR Green Supermix (Bio-Rad Laboratories, Korea) in qPCR (Bio-Rad Laboratories). The qPCR conditions were as follows: 95°C for 10 min (pre-denaturation) and 40 cycles of two steps, i.e., 95°C for 15 s (denaturation) and 60°C for 1 min (annealing and extension). Each sample was performed in duplicate, and the acceptable standard deviation (SD) of the duplicate threshold cycle (∆Ct) values was set at 0.7. The run was repeated in cases of unacceptable SD values. The relative mtDNA copy number was calculated using the equation 2^−∆∆Ct^ (∆Ct = Ct_mtDNA CYTB_ − Ct_PK_) according to a previous report (32).

### Bisulfite Modification of DNA and Methylation-Specific Polymerase Chain Reaction (MSP)

Genomic DNA (200–500 ng) was prepared for bisulfite conversion according to the manufacturer’s instructions (EpiJET Bisulfite conversion kit). The technique was based on bisulfite treatment of genomic DNA, which converts all unmethylated cytosines to uracils, whereas methylated cytosines remain as cytosines. The bisulfite-converted DNA was used as the template for methylation-specific polymerase chain reaction (MSP) to determine the DNA methylation state of *PPARGC1A* promoter and D-loop region of mitochondria. The methylated or unmethylated DNA was quantified using primers of the *PPARGC1A* promoter and D-loop regions, as described by Sookoian et al. (33) and Zheng et al. (34), respectively (33, 34). The primer sequences were as follows: for the methylated DNA of the *PPARGC1A* promoter, forward primer 5’-ATTTTTTATTGTTATGGGGGTAGTC-3’ and reverse primer 5’-AAAAATATTTAAAAACGCAAACGAA-3’; for the unmethylated DNA of *PPARGC1A* promoter, forward primer 5’-TTTTATTGTTATGGGGGTAGTTGA-3’ and reverse primer 5’-AAAAAATATTTAAAAACACAAACAAA-3’; for the methylated DNA of D-loop region, forward primer 5’-TAGGAATTAAAGATAGATATTGCGA-3’ and reverse primer 5’-ACTCTCCATACATTTAATATTTTCGTC-3’; and for the unmethylated DNA of D-loop region, forward primer 5’-GGTAGGAATTAAAGATAGATATTGTGA-3’ and reverse primer 5’-ACTCTCCATACATTTAATATTTTCATC-3’. The PCR conditions are presented in **Table S1**. The qPCR (Bio-Rad Laboratories) was performed using IQ SYBR Green Supermix (Bio-Rad Laboratories). Each sample was performed in duplicate using 20 ng converted DNA per 10 µl reaction. The acceptable SD of the ∆Ct values was set at 0.7. The level of methylated DNA was expressed as the ratio of the estimated amount of methylated DNA to unmethylated DNA level and calculated for each sample using the equation 2^−∆∆Ct^ (∆Ct = Ct_methyl_ − Ct_unmethyl_).

### Statistical Analyses

Independent *t*-tests for continuous variables and chi-square or Fisher’s exact tests for categorical variables were used for comparison of the demographic and clinical characteristics between the ADHD and HC groups.

Comparison of the mtDNA copy number, the methylation ratio of *PPARGC1A* gene promoter, and the methylation ratio of the D-loop region between the ADHD and HC groups was performed using Mann–Whitney *U* tests and also among ADHD subtypes using Kruskal–Wallis tests because the variables did not follow a normal distribution. As there was a significant difference in IQ score between the ADHD and HC groups, we applied ranked ANCOVA to compare the mtDNA copy number, the methylation ratio of *PPARGC1A* gene promoter, and the methylation ratio of the D-loop region between the two groups. Linear regression analyses were conducted to investigate the association between mitochondrial biomarkers and ADHD-RS variables, with IQ as a covariate. All statistical analyses were performed using SPSS (ver. 22.0; SPSS Inc., Chicago, IL, USA) and Prism 7 software (GraphPad Software, Inc., La Jolla, CA, USA). Statistical significance was set at a probability level of *p* < 0.05.

## Results

**Table S2** contains the demographic and clinical characteristics of the 263 ADHD patients and 99 HCs. Significant differences in age and gender existed between the ADHD and HC groups (*p* < 0.001), but these were compensated for in the analyses by our age- and gender-matched sample. The demographic and clinical characteristics of the age- and gender-matched ADHD and HC groups are presented in **Table 1**. The IQ level was lower in ADHD patients than in HCs, but no differences in age, gender, maternal and paternal education levels, or family income were observed between the two groups.

**Table 1:** Demographic and clinical characteristics of the age- and gender-matched attention-deficit hyperactivity disorder (ADHD) and healthy control (HC) groups.

Characteristic	ADHD (*n* = 70)	HC (*n* = 70)	*p* value
Age (years), mean (SD)	9.8 (2.6)	10.0 (2.6)	0.650
Sex (male), *N* (%)	44 (62.9)	44 (62.9)	1.000
IQ, mean (SD)	105.7 (12.9)	114.8 (12.1)	<0.001
Paternal education, years, mean (SD)	14.8 (1.9)	14.6 (2.6)	0.546
Maternal education, years, mean (SD)	14.7 (2.2)	13.2 (3.5)	0.696
ADHD subtype, *N* (%)			
Inattentive	25 (35.7)		
Hyperactive–impulsive	22 (31.4)		
Combined type	14 (20)		
NOS	8 (11.4)		

ADHD, attention-deficit hyperactivity disorder; HC, healthy control; IQ, intelligence quotient; NOS, not otherwise specified; NS, non-significant.

**Figure 1:**
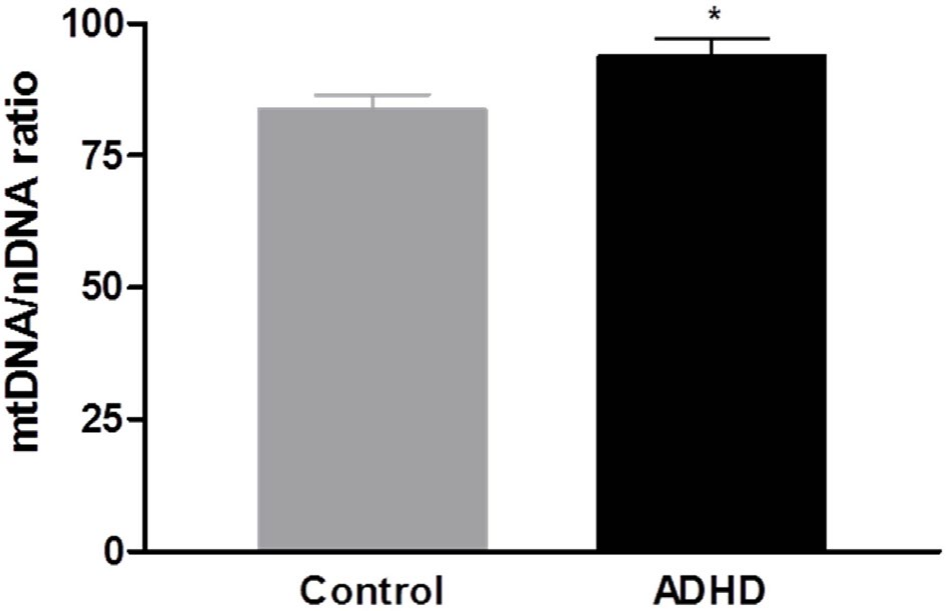
Comparison of the mitochondrial DNA (mtDNA) to nuclear DNA (nDNA) ratio in peripheral blood between attention-deficit hyperactivity disorder (ADHD) and control groups.*p < 0.05.

**Figure 2:**
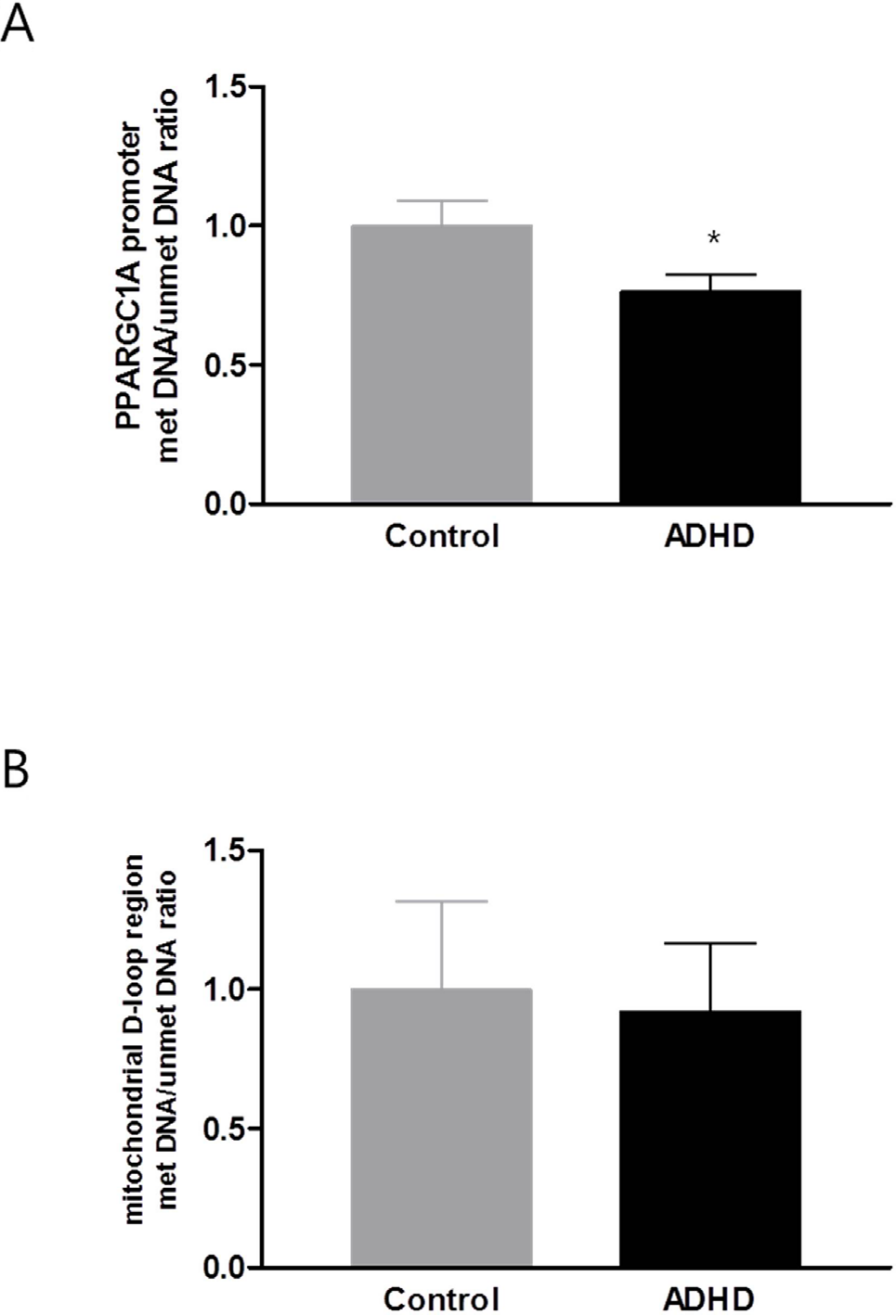
DNA methylation level of *PPARGC1A* promoter region **(A)** and D-loop region **(B)**. *PPARGC1A*, peroxisome-proliferator-activated receptor γ co-activator-1α; ADHD, attention-deficit hyperactivity disorder. *p < 0.05.

The mtDNA copy number in the blood samples was significantly increased in the ADHD group compared with the HC group (**p** = 0.028; **Figure 1**). In the DNA methylation ratio study, the ADHD group showed a lower methylation ratio than the HC group at the *PPARGC1A* promoter region (*p* = 0.008; **Figure 2A**). No significant difference was observed in the DNA methylation ratio of the D-loop region between the ADHD and HC groups (**Figure 2B**). After adding IQ as a covariate, the statistical difference of the mtDNA copy number between the two groups remained significant (*p* = 0.01), but there was no significant difference in the DNA methylation ratio of the *PPARGC1A* promoter region and the D-loop region.

There were no significant differences in mtDNA copy number and the methylation ratio of the D-loop region among subtypes in the ADHD group (*p* = 0.894 and *p* = 0.802, respectively). There was a significant difference in the methylation ratio of the *PPARGC1A* promoter region among ADHD subtypes (*p* = 0.030; inattentive subtype 0.17 ± 0.09, hyperactive–impulsive subtype 0.12 ± 0.03, combined subtype 0.23 ± 0.12, not-otherwise specified subtype 0.20 ± 0.12). These results remained significant when excluding the not-otherwise specified subtype (*p* = 0.019). No significant associations were observed between mitochondrial biomarkers and variables of ADHD-RS (**Table 2**).

**Table 2:** Correlation between mtDNA copy number, methylation ratio of *PPARGC1A* and D-loop region with ADHD-RS subscores in ADHD patients.

	mtDNA		Methylation ratio of *PPARGC1A*		Methylation ratio of D-loop	
	**Beta**	***p*** ** value**	**Beta**	***p*** ** value**	**Beta**	***p*** ** value**
ADHD-RS subscore						
Inattention	−0.156	0.809	0.001	0.808	−0.012	0.454
Hyperactivity–impulsivity	−0.836	0.126	0.002	0.342	0.003	0.804
Total	−0.383	0.271	0.001	0.462	−0.002	0.803

mtDNA, mitochondria DNA; PPARGC1A, peroxisome-proliferator-activated receptor γ co-activator-1α; ADHD-RS, ADHD Rating Scale IV; ADHD, attention-deficit hyperactivity disorder.

## Discussion

In the present study, mtDNA copy numbers were compared for the first time between ADHD patients and healthy youth. The mtDNA copy number was increased in the ADHD group compared with the HC group, even after adjusting for IQ. Regulation of mtDNA copy number is essential for cells to meet their energy requirements, especially for those requiring high energy such as neurons. Therefore, an increased mtDNA copy number may be induced by a compensatory mechanism against mitochondrial dysfunction arising from genetic and/or environmental causes (20). The relative mtDNA copy number did not show any significant associations with the variables of the ADHD-RS, possibly due to the lack of adequate statistical power to detect an association, or because the mtDNA copy number has no direct impact on the clinical features of ADHD. Further studies with larger samples are warranted.

Recent studies on the role of mitochondrial functions in neurodevelopmental disorders have focused on autism spectrum disorder (ASD). In several reports, the mtDNA copy number was increased in the peripheral blood of ASD patients (32). Bioenergetic crisis during brain development, and mtDNA mutation or deletion, has been previously suggested to cause neurodevelopmental disorders (15); the present study may contribute to the mitochondria hypothesis regarding neurodevelopmental disorders. Studies on mitochondrial genetic brain function are scarce. Heteroplasmic mice (which refers to generation of mice containing an equal mixture of two different types of mtDNA) exhibited impaired memory retention capacity (35). These results suggests connection between subtle changes in mtDNA and broad effects on brain function.

One mechanism leading to increased mtDNA copy number is increased oxidative stress (36). Although studies on oxidative stress in ADHD have been inconsistent, several studies have reported elevated oxidative stress in ADHD patients (37–39). Increases in oxidant levels could be linked to the pathophysiology of ADHD by impairment of dopamine structure and function (40). For example, mitochonridal dysfunction could lead to elevated levels of hydrogen peroxide (H_2_O_2_), which suppresses striatal dopamine release (41), leading to the dopamine deficiency found in ADHD. Dopamine is easily oxidized and generates highly reactive metabolites, such as dopamine quinine, causing a viscous cycle of further mitochondrial dysfunction and oxidative stress (42).

In the present study, decreased methylation at the promoter region of *PPARGC1A* in ADHD patients compared with controls was reported for the first time. The *PPARGC1A* controls the production of mitochondrial proteins and is considered the master regulator of mitochondrial biogenesis (43). Recently, *PPARGC1A* has been implicated in dopamine neuronal function and viability (44). However, there was no difference in the level of methylation of *PPARGC1A* after adjustment for IQ. Defective mitochondrial energy production and the resulting increased levels of free radicals have been indicated to be culprits in intellectual-disability-related diseases like Down, Rett, and Fragile X syndrome (45). Although individuals with intellectual disability were excluded from our study, our results suggest that methylation ratio of the* PPARGC1A* is associated with IQ raher than ADHD per se. The D-loop region is critical in controlling the replication of mtDNA and transcription and organization of the mitochondrial nucleoid (46). The methylation ratio of the D-loop region was not different between the ADHD patients and HCs. This may be due to the small sample size or to a possible lack of cytosine methylation in the mitochondrial genome; in a previous study, an artifact of mtDNA bisulfite sequencing was identified that can lead to an overestimation of mtDNA methylation levels (47).

This study had several limitations. First, the relatively small sample size may have limited the statistical power of the results. Second, we included all subtypes of ADHD due to the limited sample size so that the study group was heterogeneous in terms of various behavioral characteristics. Moreover, there was a significant difference in the methylation ratio of the *PPARGC1A* gene promoter among ADHD subtypes. Further studies with larger sample sizes are warranted to clarify the effect of subtypes on the results. The case-control study design may also have resulted in population stratification. Moreover, we analyzed peripheral blood, whereas brain tissues are considered the standard target tissues for psychiatric disorders. However, the mtDNA copy number in peripheral blood was shown to correlate with the mtDNA copy number in brain tissues in mice (48). Further studies investigating the change of mitochondrial biomarkers in brain tissues are warranted to confirm the results of this study. In addition, MSP was performed with just one methylation site in the promoter region of the *PPARGC1A* gene and D-loop region, and this method could not encompass the other methylation sites and mitochondrial biogenesis-related genes. Finally, our study only included Korean children and adolescents, which may limit the generalizability of the results to other ethnic populations.

In sum, we observed an elevated mtDNA copy number in ADHD patients compared with HCs. The results from the present study indicate the possible involvement of mitochondrial dysfunction in the pathophysiology of ADHD. Further studies with larger cohorts investigating the correlation between clinical markers and biomarkers of mitochondrial dysfunction are warranted to help further elucidate the clinical role of mitochondrial dysfunction in ADHD.

## Ethics Statement

Written informed consent was obtained from all parents, guardians, and adolescents, and the children provided verbal consent to participate after sufficient explanation of the study prior to enrollment. All study protocols were approved by the Institutional Review Board of Seoul National University Hospital, Seoul, Korea.

## Author Contributions

J-WK, SAK, and B-NK designed the experiment, and S-YL, MP, and SYK performed the experiment and drew the figures. JK analyzed the data and wrote the manuscript. All authors reviewed the manuscript and finally approved the version to be published.

## Funding

This research was supported by the Basic Science Research Program through the National Research Foundation of Korea (NRF) funded by the Ministry of Science, ICT, and Future Planning (NRF-2015R1A2A2A01004501 to J-WK); by the Promising-Pioneering Researcher Program through Seoul National University (SNU) in 2015 to J-WK; by a National Research Foundation of Korea (NRF) grant funded by the Korean government (MSIP, No. 2015M3C7A1028926 to B-NK and MOE, No. 2017R1D1A3B03033533 to SAK): and by the Bio & Medical Technology Development Program of the National Research Foundation (NRF) & funded by the Korean Government (MSIT) (2019M3E5D1A01069345).

## Conflict of Interest Statement

The authors declare that the research was conducted in the absence of any commercial or financial relationships that could be construed as a potential conflict of interest.
