# Supplementary material for: Peripheral Mitochondrial DNA Copy Number is Increased in Korean Attention-Deficit Hyperactivity Disorder Patients
Source: Front Psychiatry. 2019 Jul 18;10:506. doi: 10.3389/fpsyt.2019.00506 (PMC6656858; doi:10.3389/fpsyt.2019.00506)
Supplement: Supplementary file 1 [file DataSheet_1.docx]

Supplementary Table S1. MSP conditions for *PPARGC1A* promoter and D-loop region

|  | | Pre-  denaturation | denaturation | Annealing  and extension | cycles |
| --- | --- | --- | --- | --- | --- |
| *PPARGC1A*  promoter | methyl | 95ºC  for 10 min | 95ºC  for 15 sec | 59.7ºC for 1 min | 40 cycles |
|  | unmethyl |  |  | 57ºC for 1 min |  |
| D-loop  region | methyl |  |  | 60ºC for 1 min |  |
|  | unmethyl |  |  | 59.7ºC for 1 min |  |

Abbreviations: PCR, polymerase chain reaction; MSP, methylation-specific polymerase chain reaction; *PPARGC1A*, peroxisome-proliferator-activated receptor γ co-activator-1α

Supplementary Table S2. Demographic and clinical characteristics of the ADHD and HC groups before matching based on age and gender

| Characteristic | ADHD (n = 263) | HC (n = 99) | *p*-value |
| --- | --- | --- | --- |
| Age (years), mean (SD) | 8.9 (2.4) | 10.5 (2.9) | **<0.001** |
| Sex (male), N (%) | 191 (77.6) | 52 (54.7) | **<0.001** |
| IQ, mean (SD) | 105.9 (14.5) | 114.0 (11.8) | **<0.001** |
| Yearly family income >$2500, N (%) | 143 (71.9) | 56 (63.6) | 0.164 |
| Paternal education, years, mean (SD) | 14.9 (1.9) | 14.6 (2.0) | 0.306 |
| Maternal education, years, mean (SD) | 14.6 (2.3) | 14.2 (3.2) | 0.369 |
| ADHD subtype, N (%) |  |  |  |
| Inattentive | 92 (37.4) |  |  |
| Hyperactive-impulsive | 15 (6.1) |  |  |
| Combined type | 113 (45.9) |  |  |
| NOS | 26 (10.6) |  |  |

Abbreviations: ADHD, attention-deficit hyperactivity disorder; HC, healthy control; SD, standard deviation; IQ, intelligence quotient; NOS, not otherwise specified
